# Should We Maintain Anticoagulation after Successful Radiofrequency Catheter Ablation of Atrial Fibrillation? The Need for a Randomized Study

**DOI:** 10.3389/fcvm.2017.00085

**Published:** 2017-12-21

**Authors:** Giuseppe Santarpia, Salvatore De Rosa, Jolanda Sabatino, Antonio Curcio, Ciro Indolfi

**Affiliations:** ^1^Division of Cardiology, Department of Medical and Surgical Sciences, Magna Græcia University, Catanzaro, Italy; ^2^URT-CNR, Department of Medicine, Consiglio Nazionale delle Ricerche, Catanzaro, Italy

**Keywords:** anticoagulation, radiofrequency catheter ablation, atrial fibrillation, thromboembolic risk, bleeding risk

## Abstract

**Background:**

Atrial fibrillation (AF) is associated with a high risk of thromboembolic stroke and oral anticoagulation therapy (OAT) is able to reduce the rate of ischemic events. Nevertheless, the actual benefit of prolonged OAT after successful radiofrequency catheter ablation (RFCA) is not clear yet.

**Methods:**

Scientific investigations were assumed suitable if they assessed the clinical significance of the use of anticoagulation versus no anticoagulation in AF patients undergoing successful RFCA. The odds ratio (OR) with 95% confidence interval (CI) was used as the study summary measure.

**Results:**

At meta-analysis, the rate of total thromboembolic events was not significantly different between the groups (OR 1.83, 95% CI 0.69–4.88; *p* = 0.221), while a lower incidence of total bleeding events in patients not treated with OAT was found (OR 6.5, 95% CI 1.93–21.86; *p* = 0.002).

**Conclusion:**

This meta-analysis raises doubts about the net clinical benefit (NCB) of a long-term prophylactic OAT in patients with AF underwent to successful RFCA. In fact, despite similar rate of thromboembolic events, the apparent increase in bleeding risk suggests caution in prolonging OAT after RFCA. However, the lack of prospective randomized studies does not allow a comprehensive appraisal of this issue. Thus, we propose the design of a novel prospective randomized trial to evaluate the NCB of prolonged OAT after successful RFCA of AF.

## Background

Atrial fibrillation (AF) is associated with a higher rate of cerebrovascular ischemic accidents (1). Hence, oral anticoagulation represents a cornerstone in the clinical management of AF patients to reduce the risk of thromboembolic events (2). In fact, oral anticoagulation therapy (OAT) was able to achieve a 60% relative risk reduction of ischemic stroke, compared to placebo (3). For this reason, clinical guidelines for the management of AF suggest the use of OAT in all AF patients with high thromboembolic risk, with the use of percutaneous left atrial appendage occlusion being recommended in patients with clear contra-indications for OAT (4–6). At the same time, radiofrequency catheter ablation (RFCA) seems to be very effective for the treatment of AF, showing a success rate ranging from 79.7 to 88.6% for paroxysmal AF and from 66.1 to 80% for the persistent form (7). Although the indication for anticoagulation therapy in AF patients is based on their thromboembolic risk (8–11), it is not clear what the correct behavior regarding anticoagulation management in AF patients undergoing a successful RFCA should be. Current guidelines recommend life-long extension of OAT even after successful RFCA in case of high thromboembolic risk, while no clear recommendations are provided for patients at low thromboembolic risk (4–6). In fact, the lack of randomized trials testing long-term safety and efficacy of OAT in this specific population makes clinicians unsure on their actual usefulness in AF patients after successful RFCA. In this setting, we performed a critical revision of available clinical data on the advantage of anticoagulation versus no anticoagulation in AF patients after successful RFCA, adopting a meta-analytic approach, to integrate the conclusions of all eligible academic works with the following definite objectives: (1) evaluate safety and efficacy of anticoagulation versus non-use of anticoagulation in AF patients undergoing successful RFCA and (2) highlight the current knowledge gaps and design a randomized trial to test safety and efficacy of anticoagulation in AF patients undergoing successful RFCA, without selection bias.

## Methods

### Search Strategy and Study Selection

A comprehensive literature search was performed in November 2016, using PubMed and Google Scholar electronic databases, for all articles comparing OAT to no oral anticoagulation therapy (non-OAT), published in the English language in peer-reviewed journals. We excluded case reports, review articles, expert opinions, and letters to the editor. The search was performed, with no limit to the year of publication, using the following keywords: “atrial fibrillation,” “anticoagulation,” and “radiofrequency catheter ablation.” All items were screened by two researchers for eligibility, independently. To minimize the risk of overseeing relevant studies, also references of selected articles were screened, as previously described (12). All selected studies were entirely checked and classified to exclude duplicity of data. Screened studies were included if they: (a) compared the clinical outcome with anticoagulation versus no anticoagulation in AF patients after successful RFCA; (b) reported numerical outcome data [stroke, transient ischemic attack (TIA) or systemic embolism (SE), bleedings]. Reasons for exclusion were: duplicate publication, the above listed endpoint were not reported. Study quality assessment was performed as previously described and according to the PRISMA statement (12, 13).

### Data Abstraction and Quality Assessment

**Figure 1:**
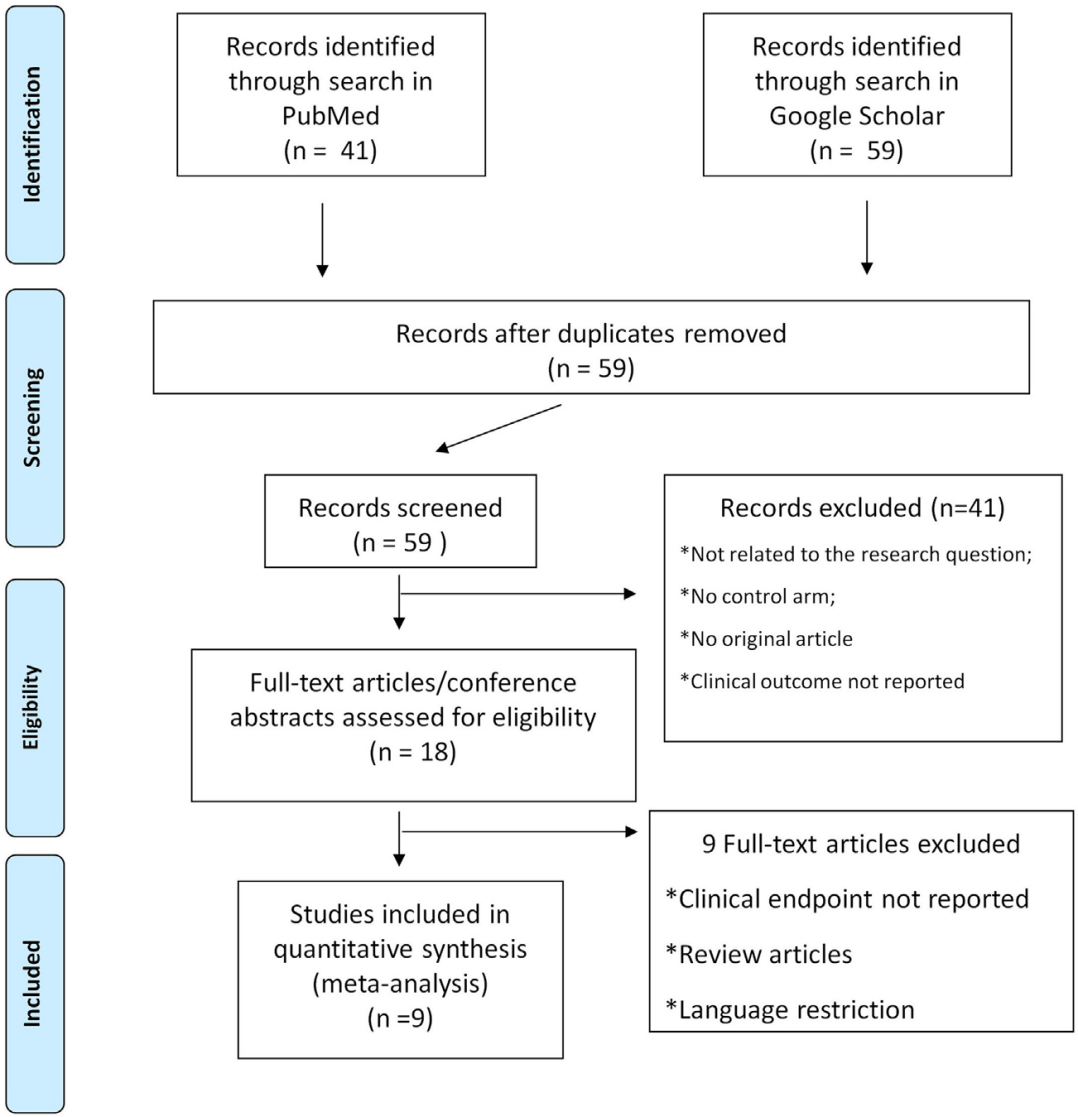
Study selection flow chart. Selection and data abstraction process, performed according to the PRISMA statement.

The titles, abstracts, and selected full texts generated from the literature search were independently screened by two authors. Data from the studies that met all inclusion criteria were manually extracted and entered into a standard extraction table. All selected articles were reviewed to extract all relevant data: publication year, study origin, number of patients included, type of study design, outcome data reported, key baseline variables. Selection and data abstraction were performed as previously described (Figure 1) (12, 13).

### Statistical Analysis

We used the odds ratio (OR) with 95% confidence interval (CI) as the summary measure. The meta-analysis was calculated using the random-effects model (14). In addition, the Mantel–Haenszel method (fixed effect model) was used alternatively, if required (15). Meta-analysis results were displayed using Forest plots, as described elsewhere (16). Heterogeneity was evaluated by means of the Cochrane Q test (*p* < 0.10 considered significant). *I*^2^ values were also calculated, with a pre-specified *I*^2^ threshold of 20%. The random-effects model was used preferentially, where appropriate (17). Funnel plots were used to assess the eventual impact of small study effects (Figure S1 in Supplementary Material). Analyses were performed using Excel spreadsheets and OpenMetaAnalyst-0.1.

## Results

### Study Characteristics

Database search produced 41 entries from PubMed and 59 from Google Scholar. In total, 59 studies were available after removal of duplicates. Of these, 41 studies were excluded according to the pre-specified criteria. Finally, nine studies were selected for meta-analysis (18–26). The stepwise selection process is drawn in Figure 1, while details of the selected studies are displayed in Table 1. Of all selected studies, five are single-center retrospective analyses (19–23) and four are multi-center retrospective, non-randomized, observational studies (18, 24–26) evaluating the safety and efficacy of oral anticoagulation during the follow-up, after a successful RFCA of AF. Quality assessment and risk of bias of the studies included in the analysis are shown in Table S1 in Supplementary Material. Briefly, no blinding was used in any of the studies included. Foremost, an evident selection bias emerges from the bias assessment. In fact, baseline thromboembolic and bleeding risk profiles were not balanced between the study arms for most of the studies included in the analysis and heterogeneously reported. Information on baseline CHADS_2_ or the CHA_2_DS_2_-VASc scores are reported in Table 1, while events recorded in each study included in the meta-analysis are displayed in Table 2; Table 3 summarizes endpoint definitions of the included studies. Long-term anticoagulation was reintroduced after the RFCA and kept for 3–12 months; it was represented by adjusted-dose warfarin in eight studies and either by warfarin or by non-vitamin K antagonist oral anticoagulants (NOACs) in the remaining study (22).

**Table 1:** Baseline patients’ risk across the studies.

Study	Nationality	Study subgroup	*N*	Thromboembolic risk score	Therapy
Bunch et al. (19)	USA	Oral anticoagulation therapy (OAT)	507	CHADS_2_score ≥ 2; 54.8%	Warfarin (INR 2–3)
		Non-OAT	123	CHADS_2_score ≥ 2; 0	ASA
Karasoy et al. (24)	Denmark	OAT	2476	CHA_2_DS_2_-VASc score ≥ 2; 37.2%	Warfarin (INR 2–3)
		Non-OAT	1574		None
Oral et al. (28)	USA	OAT	357	–	Warfarin (INR 2–3)
		Non-OAT	398	–	None
Saad et al. (21)	USA–Brazil	OAT	22	CHADS_2_score ≥ 2; 71%	Warfarin (INR 2–3)
		Non-OAT	293		ASA/None
Themistoclakis et al. (25)	USA–Italy–France	OAT	663	CHADS_2_score ≥ 2; 37%	Warfarin (INR 2–3)
		Non-OAT	2692	CHADS_2_score ≥ 2; 13%	ASA
Uhm et al. (18)	Korea	OAT	312	CHA_2_DS_2_-VASc score ≥ 2; 44%	Warfarin (INR 2–3)
		Non-OAT	296	CHA_2_DS_2_-VASc score ≥ 2; 41%	ASA
Winkle et al. (22)	USA	OAT	48	CHADS_2_score ≥ 2; 100%	Warfarin(INR 2–3)/NOACs
		Non-OAT	60		ASA/None
Yagishita et al. (23)	Japan	OAT	124	CHADS_2_score ≥ 2; 16%	Warfarin (INR 2–3)
		Non-OAT	400		None
Själander et al. (26)	Sweden	OAT	815	CHADS_2_score ≥ 2; 44%	Warfarin (INR 2–3)
		Non-OAT	360		None

**Table 2:** Events’ list for each study included in the meta-analysis.

Reference	Therapy	Total embolic events	Stroke	TIA	SE	Major bleeding
Bunch et al. (19)	Warfarin (INR 2–3)	5	4	0	1	2
	ASA	0	0	0	0	0
Karasoy et al. (24)	Warfarin (INR 2–3)	36	–	–	36	63
	None	35	–	–	35	24
Oral et al. (28)	Warfarin (INR 2–3)	2	1	–	1	2
	None	0	0	–	0	0
Saad et al. (21)	Warfarin (INR 2–3)	–	–	–	–	3
	ASA/None	–	–	–	–	0
Themistoclakis et al. (25)	Warfarin (INR 2–3)	3	3	–	–	13
	ASA	2	2	–	–	1
Uhm et al. (18)	Warfarin (INR 2–3)	3	1	2	–	2
	ASA	1	1	0	–	2
Winkle et al. (22)	Warfarin (INR 2–3)/NOACs	1	0	0	1	9
	ASA/None	0	0	0	0	0
Yagishita et al. (23)	Warfarin (INR 2–3)	3	2	1	–	2
	None	0	0	0	–	0
Själander et al. (26)	Warfarin (INR 2–3)	5	5	–	–	3
	None	6	6	–	–	0

*TIA, transient ischemic attack/silent cerebral ischemia; SE, systemic embolism*.

**Table 3:** Endpoints’ definition for any study included in the meta-analysis.

Study	Major bleeding	Stroke	Thromboembolism
Bunch et al. (19)	Not available	Not available	Not available
Karasoy et al. (24)	Intracranial bleeding or bleeding from respiratory, gastrointestinal, or urinary tract	Not available	Ischemic stroke, transient ischemic attack (TIA), or peripheral artery embolism
Oral et al. (28)	Not available	Not available	Not available
Saad et al. (21)	Not available	Not available	Not available
Themistoclakis et al. (25)	Intracranial or retroperitoneal bleeding. Bleeding leading directly to death, or resulted in hospitalization or transfusion	The abrupt onset of focal neurological deficit persisting for more than 24 h	Not available
Uhm et al. (18)	Any type of hemorrhage requiring blood transfusion or intervention and bleeding with reduction of hemoglobin levels by ≥4.0 g/day	Symptomatic ischemic cerebral infarction with apparent brain lesion in imaging studies	Stroke, TIA, and any other systemic embolism (SE)
Winkle et al. (22)	Not available	Not available	Not available
Yagishita et al. (23)	Not available	The abrupt onset of focal neurological deficit	Stroke, TIA, and any other SE
Själander et al. (26)	Intracranial hemorrhage	The abrupt onset of focal neurological deficit	Not available

### Clinical Events

A total of nine studies were selected for the meta-analysis, including 11,520 patients (5,324 treated with prolonged anticoagulation and 6,196 treated without OAT). Altogether, 102 embolic events (58 in the OAT arm, 44 in the non-OAT arm) and 126 bleedings (99 in the OAT arm, 27 in the non-OAT arm) were captured.

### Stroke and Thromboembolism

**Figure 2:**
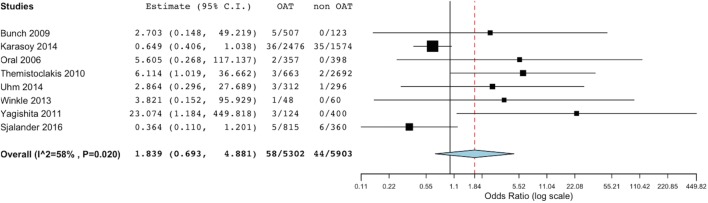
Meta-analysis of difference in total thromboembolic events. Forest plot and summary effect of the difference in the incidence of total thromboembolic events.

**Figure 3:**
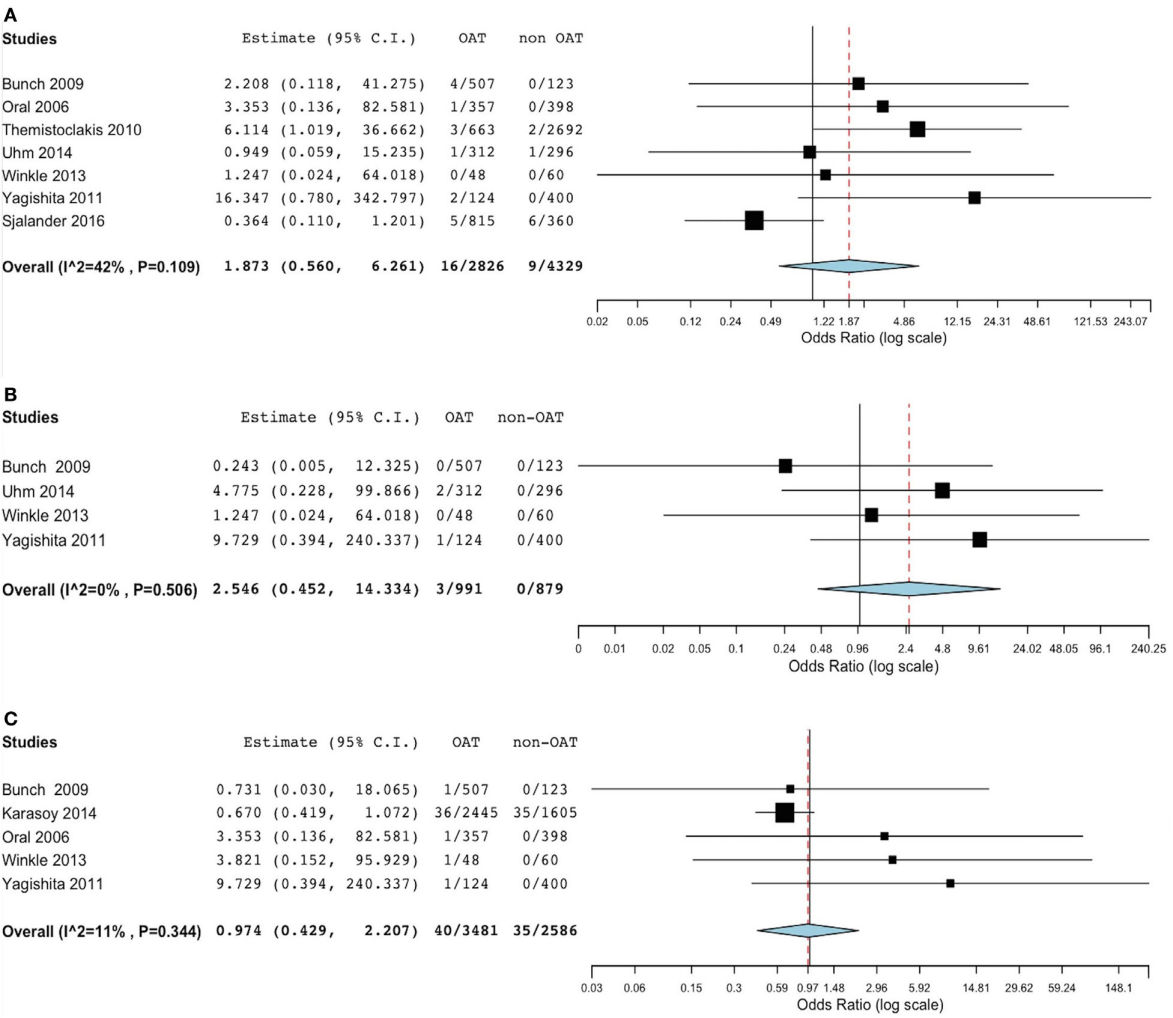
Meta-analysis of difference in stroke, transient ischemic attack (TIA) and systemic embolic events. **(A)** Forest plot and summary effect of the difference in the incidence of stroke. **(B)** Forest plot and summary effect of the difference in the incidence of TIA. **(C)** Forest plot and summary effect of the difference in the incidence of systemic embolism.

At meta-analysis, no significant difference was found in the incidence of thromboembolic events between the groups (OR 1.83, 95% CI 0.69–4.88; *p* = 0.221) (Figure 2). Of note, the sensitivity analysis revealed consistency of this result. In fact, removal of each single study did not result in significant changes of meta-analysis results. Evaluating the summary effect on the cumulative endpoint of ischemic stroke, no significant difference was evident between OAT and non-OAT-treated arms (OR 1.87, 95% CI 0.56–6.26; *p* = 0.308) (Figure 3A). A smaller number of studies independently provided numerical data on the specific endpoints of TIA and SE. Summing up the available evidence from those studies, no significant difference was found (OR 2.55, 95% CI 0.45–14.3; *p* = 0.29 for TIA and OR 0.97, 95% CI 0.43–2.2; *p* = 0.95 for SE) (Figures 3B,C).

### Bleedings

**Figure 4:**
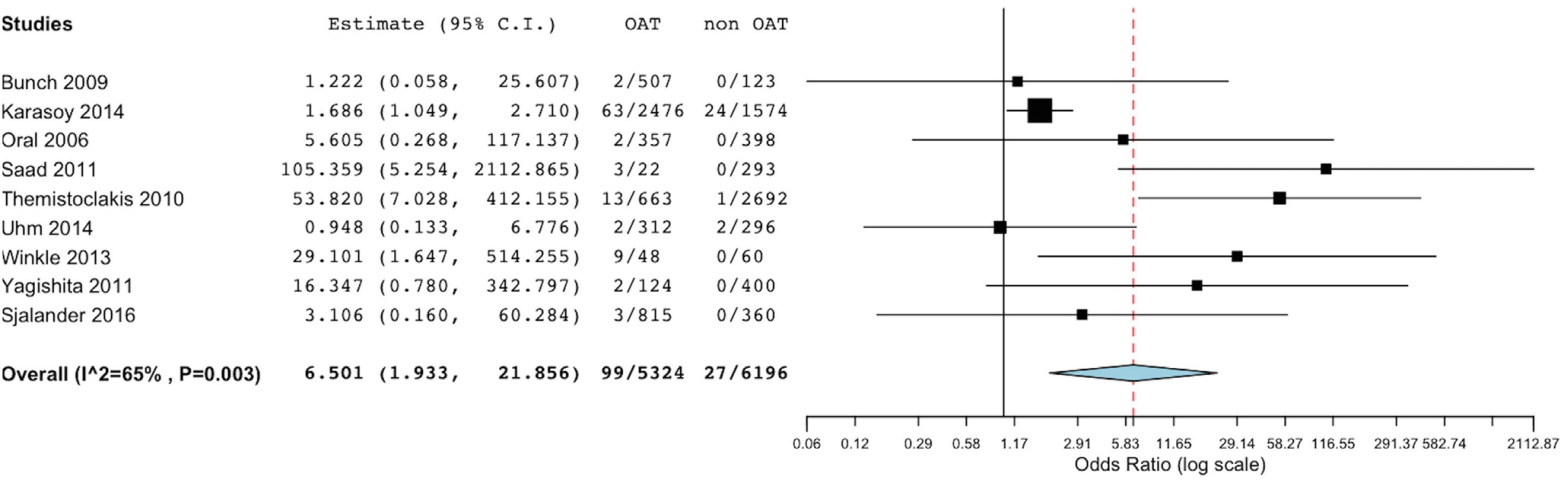
Meta-analysis of difference in total bleeding events. Forest plot and summary effect of the difference in the incidence of total bleedings.

Bleeding rates were available from all included studies. Summing up the evidence from all these studies, we found a significantly lower incidence of total bleeding events in the non-OAT arm (OR 6.5, 95% CI 1.93–21.86; *p* = 0.002) (Figure 4). At sensitivity analysis, we found consistence of the above described results across single studies, as the overall summary effect was not significantly different after alternatively removal of single studies.

## Discussion

The actual benefit of anticoagulation therapy on a medium-to long-term follow-up after successful RFCA of AF is currently unclear. In fact, the incidence of thromboembolic complications during this time window is highly variable, ranging from 0.5 to 7% (27–32). This makes it difficult to assess the impact of prolonged OAT, or the optimal duration of OAT after RFCA. None of the currently available studies on this topic is randomized and they all suffer from an obvious selection bias. In fact, baseline thromboembolic risk is unbalanced in several of the selected studies. As Oral et al. reported, thromboembolic events are generally more frequent within the 30-days time window after RFCA (20). The underlying reasons are probably multifactorial, but the almost exclusively use of warfarin in these studies may have played a role. Hence, a larger use of NOACs could improve thromboembolic prevention in the early phase. The present study is the only meta-analysis comparing prolonged OAT versus non-OAT after successful RFCA of AF. Our results suggest caution with use of prolonged OAT after successful RFCA of AF. In fact, we found a significantly lower incidence of total bleeding events in the non-OAT arm, despite no significant change in the rate of total thromboembolic events. However, given the non-randomized design of available studies, and the evident selection bias, it is difficult to discern the variations associated with OAT treatment to those related to the different baseline risk.

## Limitations of the Present Meta-Analysis

Since all studies available are non-randomized retrospective trials, we cannot exclude a sampling bias. In addition, blinded analysis or central endpoint classification and adjudication were not performed in all studies included in this meta-analysis. Accordingly, the studies were not balanced between the treatment arms. Furthermore, baseline clinical risk data were heterogeneously reported across the studies included, which made impossible to risk-stratify or to evaluate the impact of any score in meta-regression analyses to assess the impact of the observed imbalance between the treatment arms on study outcomes. These limitations are also reflected in the large CI of the effect size for most studies. Finally, bleeding events were differently classified and heterogeneously reported across the studies included in the present meta-analysis.

## Proposal for a Novel Randomized Study

**Figure 5:**
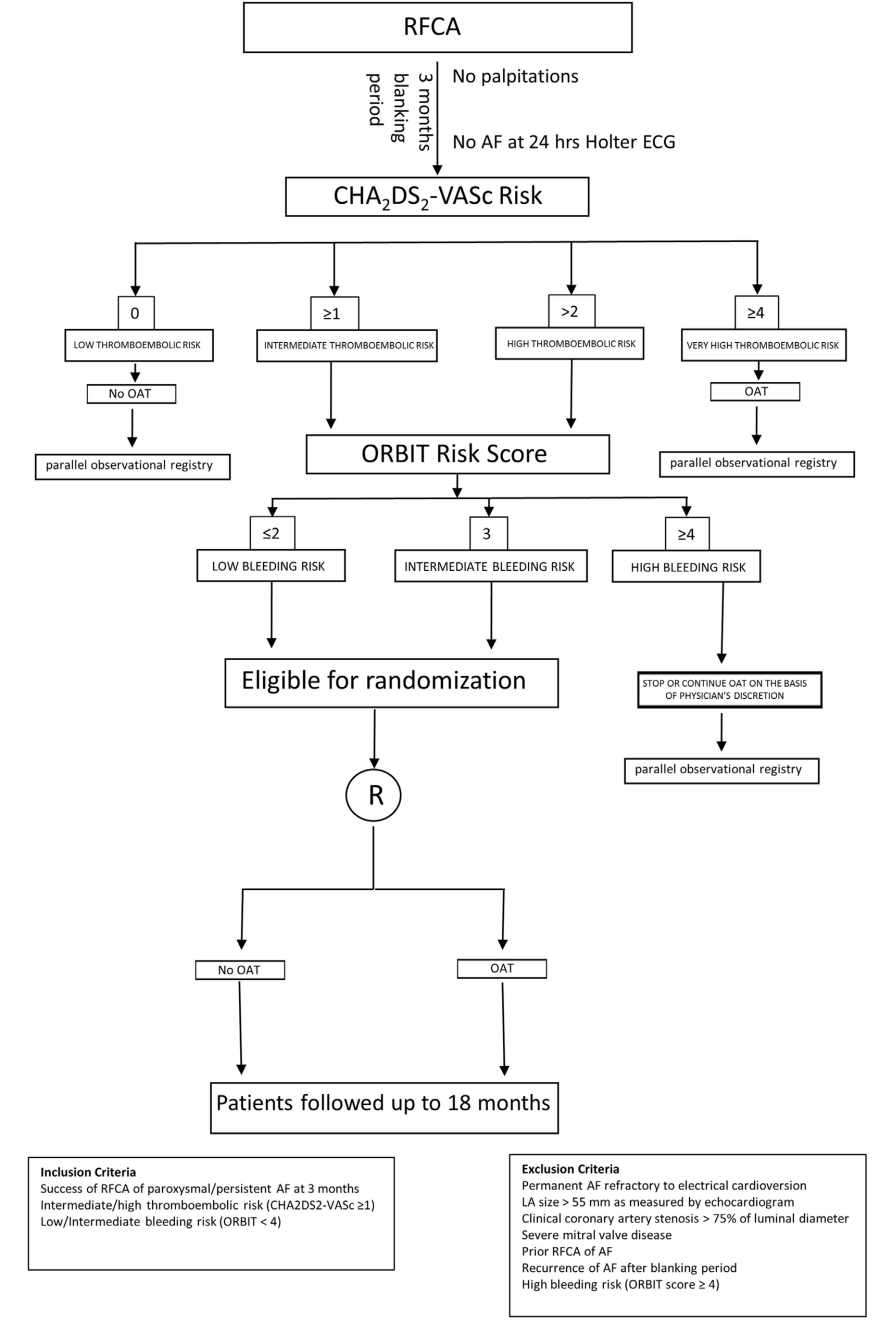

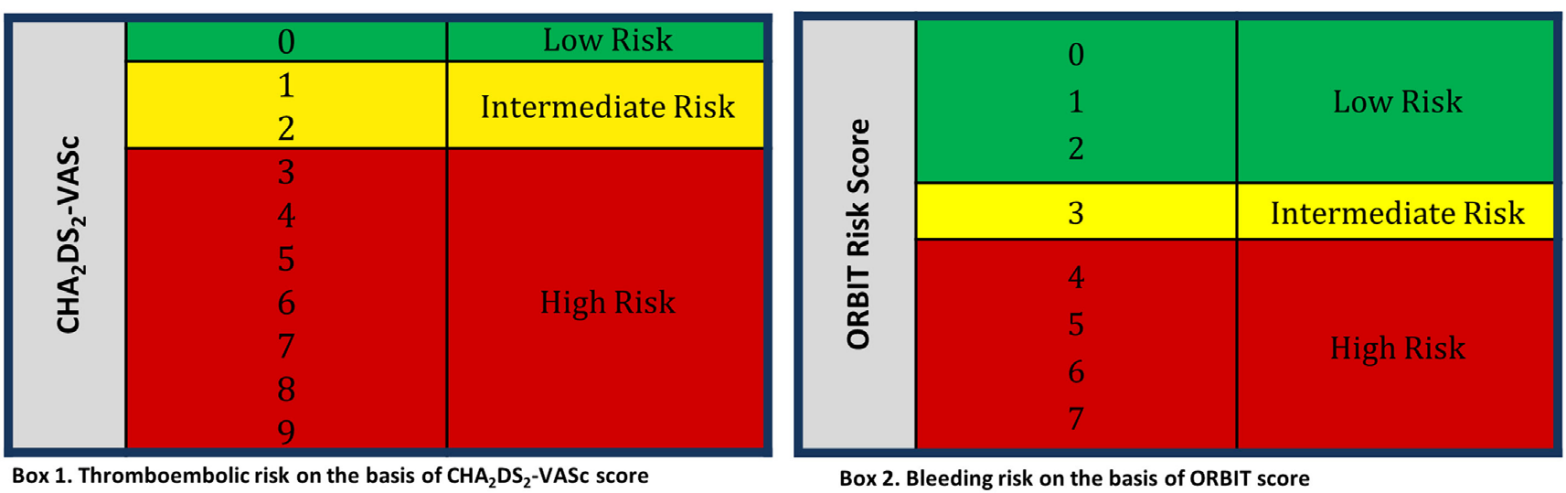
Study design flowchart. Schematic representation of patients’ selection and randomization.

**Figure 6:**
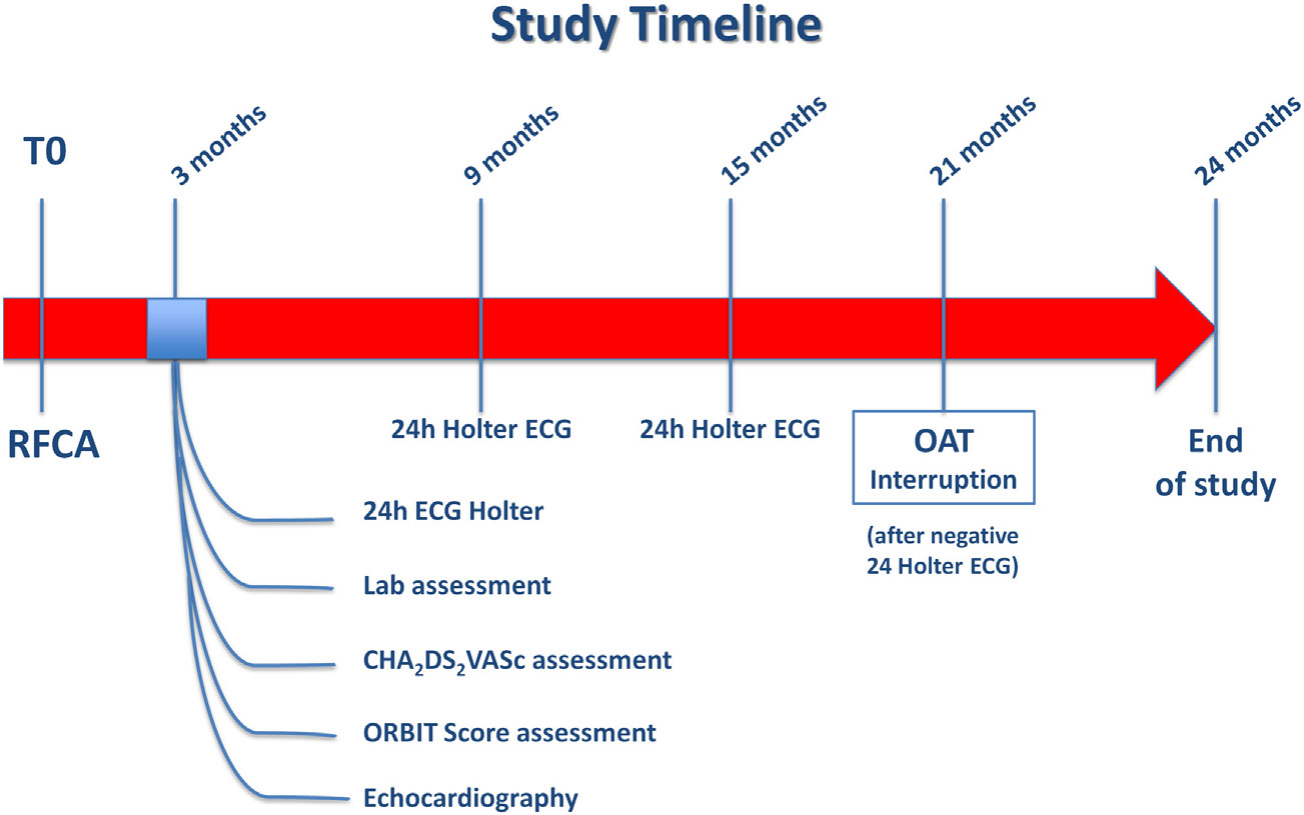
Study timeline. Study related procedures in a timeline.

In light of the significantly increased bleeding risk associated to prolonged OAT after successful RFCA of AF in the present analysis, as well as the inconclusive results on prevention of thromboembolic events from the non-randomized studies available so far, a randomized study should be designed to explore the actual benefits and risks of anticoagulation therapy in AF patients undergone to successful RFCA, as no conclusive answer to this question was found, yet. In fact, while on one hand previous and current risk stratification strategies (CHADS_2_, CHA_2_DS_2_-VASc score) seem to predict the stroke risk in AF patients at least in part independently of the presence of AF, Bunch and colleagues clearly showed that AF ablation is able to reduce the stroke risk, independently of baseline risk score (33). For this reason, eligible patients should be randomized in a 1:1 ratio to either no further anticoagulation or prolonged OAT 3 months after successful RFCA. Criteria to define a successful RFCA procedure should be lack of palpitations and absence of arrhythmia at the 24-h Holter electrocardiogram (ECG) performed 3 months after the procedure. All patients with successful RFCA, should undergo a thromboembolic risk stratification to be classified in low, intermediate or high thromboembolic risk classes using the CHA_2_DS_2_-VASc score, the most widely used and well-validated score for the assessment of thromboembolic risk in AF. Patients with CHA_2_DS_2_-VASc < 1, will be classified as low thromboembolic risk patients, while those with CHA_2_DS_2_-VASc ≥ 1will be labeled as intermediate thromboembolic risk patients and lastly, those with CHA_2_DS_2_-VASc > 2 will be included in the high thromboembolic risk patients’ category (Figure 5; Box 1). Bleeding risk will be estimated by means the ORBIT score (34), a novel and promising risk score that was specifically validated for AF patients. Eligible patients will be classified in low, intermediate, and high bleeding risk as follows: patients with ORBIT score ≤2, will be classified as low bleeding risk patients, while those with ORBIT score = 3 will be labeled as intermediate bleeding risk patients; finally, those with ORBIT score ≥4 will be included in the high bleeding risk patients’ category (Figure 5; Box 2). As reported in the study flowchart (Figure 5), patients with intermediate to high thromboembolic risk, and low to intermediate bleeding risk will be eligible for randomization to prolonged anticoagulation versus no anticoagulation therapy in 1:1 ratio. We propose to perform randomization 3 months after RFCA, to avoid inclusion of patients with early recurrence of AF. Nevertheless, presence of normal sinus rhythm at 24 h Holter ECG in asymptomatic patients does not completely exclude the possibility of AF recurrence over the mid-to long-term period. However, this reflects the situation that clinicians usually face in daily practice. In fact, a decision to prolong the OAT has to be taken at that time point, with no crystal ball to look into. Hence, the primary analysis should be performed on an “intention to treat” basis, to provide clinicians with results that should be reflecting as much as possible a real-life clinical scenario. An “as treated” analysis will additionally be performed to account for patients that will develop AF recurrence during the study period. Nevertheless, every effort should be made to detect asymptomatic AF events within the study. For this reason, we plan to repeat 24-h Holter ECG at months 9, 15, and 21. The study timeline is depicted in Figure 6. In addition, to increase the chance to detect asymptomatic AF events during the study, a subgroup of patients will be invited to participate to a sub-study (based on their age and level of confidence with mobile devices). Patients agreeing to enter the sub-study will be provided with a smartphone application to detect AF through measurement and analysis of finger-tip photopletismography. All patients will be observed for a follow-up period of 18 months after which they will enter a 3-months observational period (no OAT in both arms). Patients at low thromboembolic risk, as well as patients with very high thromboembolic risk (CHA_2_DS_2_-VASc ≥ 4) will, respectively, qualify for no anticoagulation or to anticoagulation therapy at physician discretion and they will be included in a parallel observational registry. Patients with recurrence of palpitations or documented AF during the 3 months after RFCA as well as patient with AF diagnosed by smart monitoring or 24-h Holter ECG planned during follow-up time, will qualify for OAT, irrespective of the arm they will be randomized to. Patients at high bleeding risk will be excluded. The patients’ selection and study flowchart is simplified in Figure 5. The primary endpoint will be net clinical benefit (NCB) defined as a hierarchical composite of ischemic stroke, major thromboembolic events and major bleedings. Major thromboembolic events will include systemic and pulmonary embolism, while major bleeding will be defined accordingly to the BARC bleeding classification (types 2–5) (35, 36). Secondary endpoints will be: stroke (ischemic), systemic thromboembolism, major bleedings, and non-major bleedings. Based on currently available data on the incidence of thromboembolic and bleeding events in similar populations, we estimate that 970 patients will be required to reach an 80% power for detection of a 50% decrease of the primary endpoint at a 5% significance level. However, the sample size will be enlarged to 1,010 patients (505 per arm) to account for a 2% crossover rate.

## Conclusion

Oral anticoagulation therapy is currently recommended by clinical practice guidelines for thromboembolic prophylaxis of AF patients at high thromboembolic risk. However, its actual benefits for long-term prophylaxis of AF patients after successful RFCA are unclear. Despite the beyond adduced possible drawbacks, the current meta-analysis, the broadest and most complete applicable up to now, indicates that long-term anticoagulation therapy is not free from risk of potential serious complications. In fact, a prolonged anticoagulation therapy in patients with AF after successful RFCA is associated to an increase of major bleeding events with no evidence of prevention of total thromboembolic complications. In this context, we propose the draft of a prospective randomized study to assess the best post-procedural management of AF patients treated with successful RFCA with the aim of assessing the actual NCB of a prolonged oral anticoagulation in these patients.

## Author Contributions

GS, SR, and JS conceived the study. JS performed all analyses. GS, SR, and JS interpreted analyses results and drafted the manuscript. AC and CI made critical revision of the manuscript. CI gave the final approval.

## Conflict of Interest Statement

The authors declare that the research was conducted in the absence of any commercial or financial relationships that could be construed as a potential conflict of interest.

## Supplementary Material

Figure S1 No apparent publication bias was evident from the funnel plots for the endpoints of thromboembolic events **(A)**, ischemic stroke **(B)**, transient ischemic attack **(C)**, and total bleeding events **(D)**. Circles represent studies. The position of each circle on the y-axis represents the standard error of the Log OR, while its position on the x-axis represents the effect size (OR).

## Abbreviations

AF, atrial fibrillation; RFCA, radiofrequency catheter ablation; CI, confidence interval; OAT, oral anticoagulation therapy; OR, odds ratio; TIA, transient ischemic attack; SE, systemic embolism; ECG, electrocardiogram; NOACs, non-vitamin K antagonist oral anticoagulants; NBC, net clinical benefit.
