# Supplementary figures and images for: Should We Maintain Anticoagulation after Successful Radiofrequency Catheter Ablation of Atrial Fibrillation? The Need for a Randomized Study
Source: Front Cardiovasc Med. 2017 Dec 21;4:85. doi: 10.3389/fcvm.2017.00085 (PMC5742595; doi:10.3389/fcvm.2017.00085)

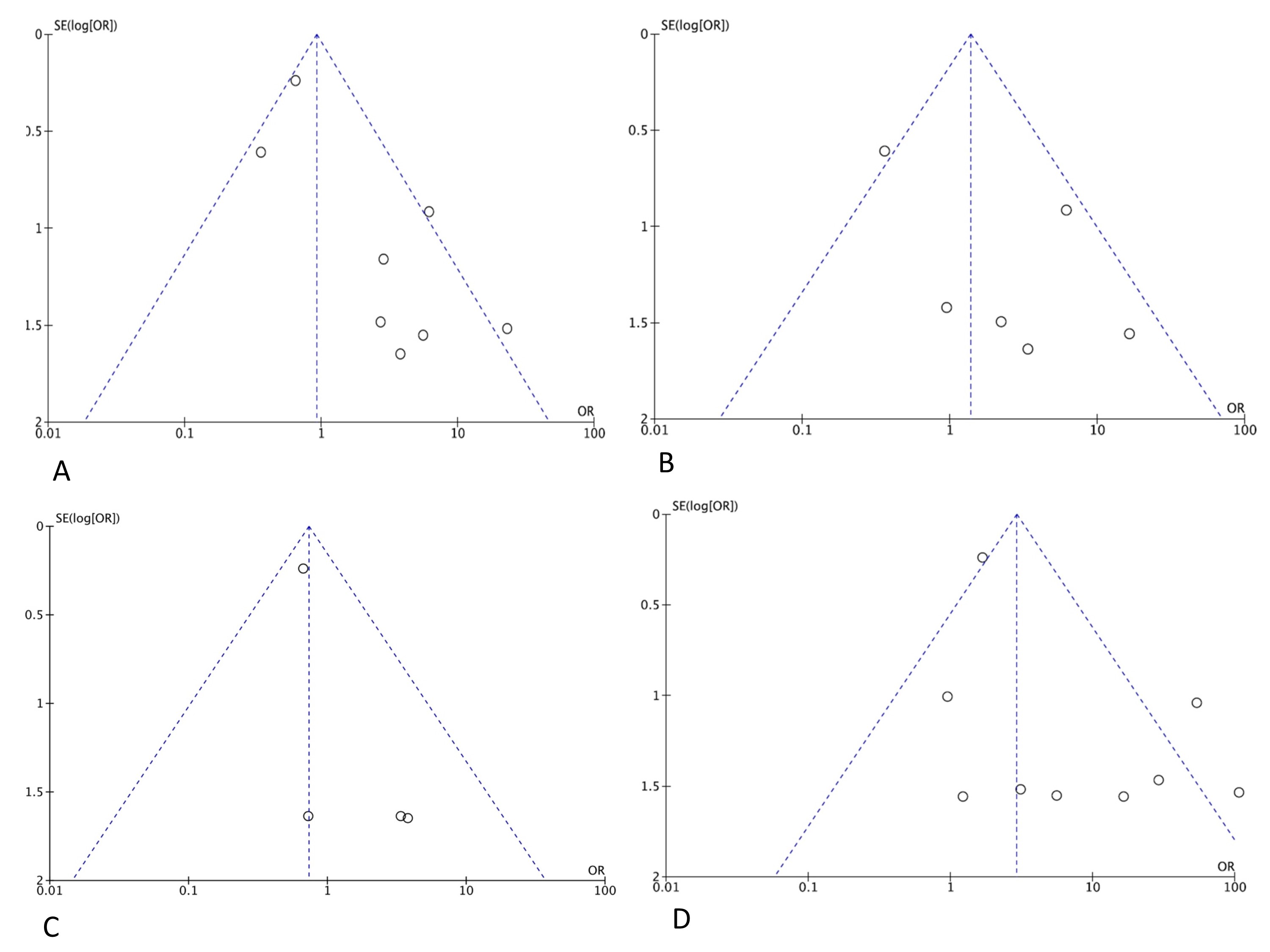

Supplement: Figure S1 — No apparent publication bias was evident from the funnel plots for the endpoints of thromboembolic events (A), ischemic stroke (B), transient ischemic attack (C), and total bleeding events (D). Circles represent studies. The position of each circle on the y-axis represents the standard error of the Log OR, while its position on the x-axis represents the effect size (OR). [file image_1.jpeg]
