# Supplementary material for: Should We Maintain Anticoagulation after Successful Radiofrequency Catheter Ablation of Atrial Fibrillation? The Need for a Randomized Study
Source: Front Cardiovasc Med. 2017 Dec 21;4:85. doi: 10.3389/fcvm.2017.00085 (PMC5742595; doi:10.3389/fcvm.2017.00085)
Supplement: Supplementary file 2 [file table_1.docx]

| **Study** | **Blinding** | **Incomplete outcome data** | **Selective outcome reporting** | **Selection bias and risk of confounders** |
| --- | --- | --- | --- | --- |
| ***Bunch*** | Not used | No (++) | Yes (++) | Baseline differences (+) |
| ***Karasoy*** | Not used | No (++) | Unclear | Yes (-) |
| ***Oral*** | Not used | No (++) | Unclear | Baseline differences (+) |
| ***Saad*** | Not used | Yes (-) | Unclear | Baseline differences (+) |
| ***Themistoclakis*** | Not used | No (++) | Yes (++) | Baseline differences (+) |
| ***Uhm*** | Not used | No (++) | Yes (++) | Baseline differences (+) |
| ***Winkle*** | Not used | No (++) | Yes (++) | Yes (-) |
| ***Yagishita*** | Not used | No (++) | Unclear | Yes (-) |
| ***Själander*** | Not used | No (++) | Unclear | Baseline differences (+) |

**Table S1. Quality assessment and risk of bias of the studies included in the meta-analysis**

- = high risk of bias; + = medium risk of bias; ++ = low risk of bias.
